# A sound case for listening

**DOI:** 10.3389/fnhum.2023.1228380

**Published:** 2023-08-03

**Authors:** Bronwyn Hoffmann, Uwe Napiersky, Carl Senior

**Affiliations:** ^1^Aston Business School, Aston University, Birmingham, United Kingdom; ^2^School of Psychology, Aston University, Birmingham, United Kingdom

**Keywords:** coaching, auditory, listening, speech, brain

## Auditory social cognition: paradox

Despite a considerable body of research dedicated to the understanding of the neural systems that underpin our ability to process speech – there is relatively scant attention given to the way sound, our evolutionary attentional system, navigates our daily interactions alerting us to potential environmental danger (Horowitz, 2012). From a neuroanatomical perspective the importance of auditory information is proposed by the auditory modality's extensive temporal lobe networking with the prefrontal cortex; suggesting more representation in the prefrontal cortex than any other sensory modality (Medalla and Barbas, 2014). Indeed, the bizarre behavioral manifestations of Paul Broca's unfortunate patient “Tan” and the subsequent discovery of a small region in the inferior frontal gyrus focused considerable research attention on the neural systems involved in the *production* of speech (Mohammed et al., 2018). However, comparatively little focus has been given over to the neurophysiological systems that are involved in *understanding* speech in general and speech that drives complex social behavior specifically and more so how the two processes interact.

That said there is interplay between the frontal regions with Broca's area and the more anterior aspects of the fronto-polar regions (e.g., Brodmann's area 10). Indeed, described as the main frontal “auditory field” the auditory input and output patterns detected in this region, suggest that the connection of auditory association cortex to the frontopolar cortex underlie the complex cognitive processes of self-reflection, prospection and forging future decisions (Medalla and Barbas, 2014). When one considers the possible neural systems that may drive the interpretation of complex and self-reflective conversation the extent of our knowledge could be conceptualized as a “listening loop” consisting of the ear (the cochlea), the primary auditory cortex and the frontotemporal regions (e.g., McAlpine and de Hoz, 2023). The paucity of our understanding is further realized when one considers the relative superficiality of the type of conversation that has been studied to date.

One only has to stop and listen to the everyday conversation to instantly realize that the speech uttered on a daily basis is very rarely the same speech that is tested in the cognitive neuroscientific laboratories around the world. Take for example the extent to which a child's speech development is scaffolded with daily exposure to a parent's interactional voice cues or “motherese” (Dodane, 2022). Here there is a direct relationship with the way that speech is processed and subsequent brain development (Nencheva and Lew-Williams, 2022). A process that is so complex that begins *in utero* and may also have an epigenetic foundation (Kisilevsky et al., 2003). Such complexity is relatively minor compared to the everyday occurrence of speech that considers the nuanced idiosyncrasies of idiolects and dialects, environmental noise as well as higher level cognitions such as the processing of metaphor etc. (e.g., Li and Zhang, 2023). In the evolution of language, Fitch (2010) illustrates how even slight differences in intonations may impact the nature of a question in conversation. Mastering the art of sound, such as tones, presents a powerful tool in building rapport, delivering impactful questions successfully traversing our complex social lives. One way to explore the cognitive neuroscience of complex auditory processing may be with the study of executive coaching which by its nature prompts a social dyad to both produce and perceive complex, higher level and reflective speech (Britten, 2015).

## What is executive coaching?

The terms executive coaching, leadership coaching, business coaching, workplace coaching, or organizational coaching are often used interchangeably (e.g., Theeboom et al., 2014; Blackman et al., 2016). We use the term executive coaching for this paper, a developmental intervention with an emphasis on helping the client learn for themselves, both personally and professionally (Athanasopoulou and Dopson, 2018). Originating in the field of sport and business, coaching's exponential growth has extended to include the fields of education and medicine (de Haan and Nilsson, 2023). Unique to coaching, is a cognitive process that is multi-layered, recursive and reflective wherein thoughts, feelings and actions are explored within a social dyad through the spoken word.

The coaching process is a unique testbed to explore the complexity of language in initiating reflective cognition. In support Darics (2019) advances linguistic awareness of subtle nuances during conversation to prompt self-reflexive management practice. In coaching, both the coach and coachee, explore core functions of the shared language network. Neuroimaging has started to elucidate the cortical systems that mediate executive coaching (Boyatzis and Jack, 2018), with the so-called “default mode network” consisting of regions such as the medial prefrontal cortex, posterior cingulate cortex as well as the angular gyrus to be implicated in some of the core coaching processes. This is perhaps to be expected when one considers that this network is often implicated with introspective processes (Medalla and Barbas, 2014). However, when one delves a little further and attempts to map the key outcomes of the coaching process to a cortical system this relationship starts to become more complex.

Additional insights on the generation of creativity/insight that may occur during a coaching session can be gained with the study of non-directive coaching (Bartolome et al., 2022). Non-directive coaching (NDC), is a client-centered conversation oriented to reflection, wherein the coach mainly observes and listens to the client speaking, mirroring what the client says, and asking open ended questions, enhancing the client's own potential for reflection (Bartolome et al., 2022). When considering the neurophysiological signature of NDC, significant activation in networks of the right parieto-temporal region occurred during the generation of (creative) insights (Bartolome et al., 2022).

## Auditory cognition and speech's social relevance: listening partners in coaching discovery

Support for the primacy of the auditory domain during executive coaching is underscored by Kluger and Itzchakov (2022) Episodic Listening Theory, in which listening induces a mutual state of creative thinking shared by dyad members. Kluger and Mizrahi (2023), recognizing excellent listening in phone conversation, propose listening be defined by dyad members' unobservable acts of devotion to each other to co-creatively explore the other. Hinz et al. (2022) echo the importance of relationships in listening, describing how knowledge is co-created during the conversational process of speaking-and-listening with others. The aforementioned findings appear to concur with what McLaughlin (2013) refers to as “the power of the aural connection” to deepen the learning process between coach and coachee during telephone coaching. Indeed, Bailenson (2021) points out, telephonic communication (auditory) has been integral in social connection for decades.

Human language and speech, is the most important medium to engage socially (Scott, 2019). According to Horowitz (2012), based on our evolutionary biology, even disparate languages, share common components of sound production, such as phonemes, morphemes and structure of words. This might explain that coaching in a second language is possible and has fewer disadvantages than expected for the coaching experience and its outcomes (Cox, 2012; de Haan, 2019). Lynden and Avery (2016) note how verbal tone, pitch and pace are crucial for building rapport between coach and coachee, to deepen coachee reflection.

The use of executive coaching as a means to study the human brain is an approach that is firmly embedded within the framework of organizational cognitive neuroscience (Senior et al., 2011). Such an approach presents the opportunity to study behavioral outcomes in response to a variety of organizational manifestations, in the natural laboratory, that is the real world. While traditional application of the organizational cognitive neuroscience approach often resides within the context of managerial behavior etc. the study of executive coaching can now be added to its portfolio (Senior et al., 2015).

## Author contributions

All authors listed have made a substantial, direct, and intellectual contribution to the work and approved it for publication.
